# The role of extracellular matrix viscoelasticity in development and disease

**DOI:** 10.1038/s44341-025-00014-6

**Published:** 2025-04-03

**Authors:** Olivia Courbot, Alberto Elosegui-Artola

**Affiliations:** 1 Cell and Tissue Mechanobiology Laboratory, The Francis Crick Institute, London, UK; 2 Department of Physics, King’s College London, London, UK

**Keywords:** Biological physics, Biophysics, Biotechnology, Cell biology, Diseases, Physics

## Abstract

For several decades, research has studied the influence of the extracellular matrix (ECM) mechanical properties in cell response, primarily emphasising its elasticity as the main determinant of cell and tissue behaviour. However, the ECM is not purely elastic; it is viscoelastic. ECM viscoelasticity has now emerged as a major regulator of collective cell dynamics. This review highlights recent findings on the role of ECM viscoelasticity in development and pathology.

## Introduction

The ECM is composed of several proteins that generate a scaffold that directly interacts with cells and tissues. Due to this interaction, cells receive not only chemical signals but also mechanical cues^1^. It has long been appreciated that the mechanical properties of the ECM regulate cell and tissue response both during development^2^ and disease^3,4^. Among the different mechanical properties of the ECM, research has focused on the influence of the elasticity of the ECM as the main determinant of tissue response. The elasticity of the ECM is generally characterised by its elastic modulus or stiffness, which is the stress (force per unit area) necessary to induce a given strain (deformation). In the late 1990s, research showed the influence of ECM stiffness in cell behaviour^5^. Since then, purely elastic materials such as polyacrylamide or polydimethylsiloxane have been widely used to study the influence of stiffness on cell and tissue responses. Research with these materials has shown that stiffness can regulate different cellular processes such as differentiation, proliferation or migration. Increased stiffness is a hallmark of several cancers, as tumours are generally stiffer than the surrounding tissue^6–9^. Cells tend to migrate more rapidly and develop a more invasive phenotype when exposed to conditions exceeding physiological stiffness^8,10,11^. Similarly, in development, stiffness has been shown to affect cell differentiation^12,13^, tissue patterning^14^ and morphogenesis^15^.

**Fig. 1:**
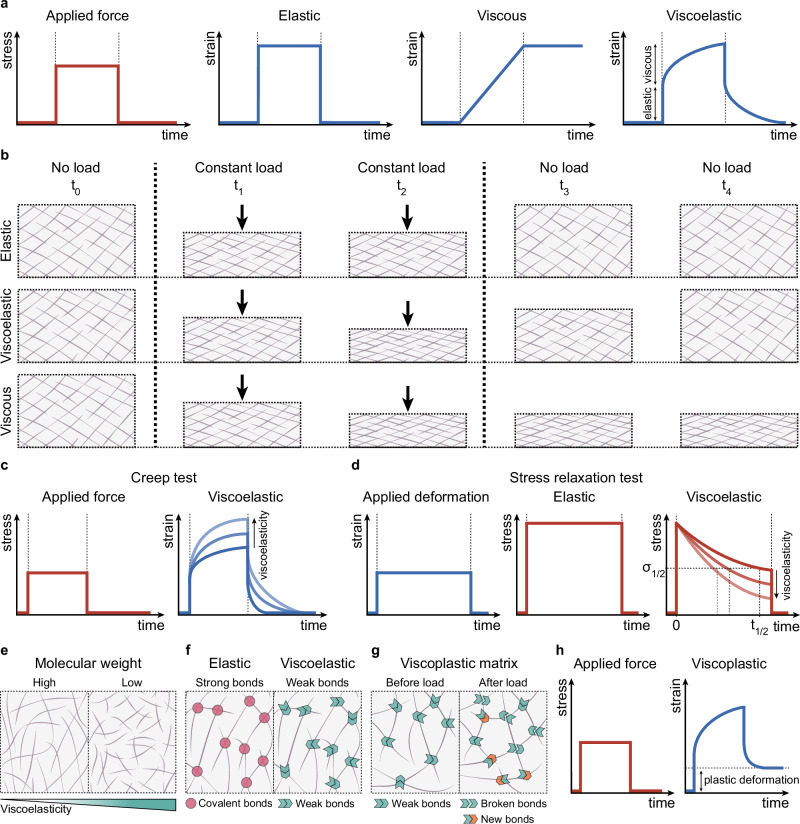
Mechanical properties of materials and relevant mechanisms that regulate ECM viscoelasticity. **a** Under a constant force, purely elastic materials deform instantly and maintain that deformation until the load is removed, at which point they immediately return to their original shape. Different from elastic materials, purely viscous materials undergo continuous deformation. Viscoelastic materials display an initial immediate response to force (elastic component) followed by an increase in the deformation over time (viscous component). Stress and strain colours are red and blue, respectively. **b** Under a constant force (black arrows), the deformation of a purely elastic matrix is time-independent: the matrix immediately deforms and returns to its initial shape when the force is applied and removed, respectively (top). Contrary to elastic matrices, viscoelastic matrices response changes with time: under a constant load, the deformation increases over time and they do not immediately return to their original shape once the load is removed (middle). Purely viscous matrices will continuously deform due to the force, and retain their deformed shape after the force is removed (bottom). **c** Creep test showing the deformation of a viscoelastic material in response to a constant force. The more viscoelastic the material is, the more it deforms over time under the load. **d** A stress relaxation test shows the evolution of the stress in the material in response to constant deformation. Purely elastic materials experience constant stress. In a deformed viscoelastic material, the stress decreases over time. The stress relaxation half-time (t_1/2_) is defined as the time needed for the stress to reach half its initial value. The more viscoelastic material is, the faster the stress decreases and, therefore, the lower t_1/2_ is. **e** Decreasing the molecular weight of the ECM components decreases the network connectivity and, therefore, increases ECM viscoelasticity, as it favours energy dissipation and fibre movements. **f** Purely elastic materials are typically crosslinked by covalent bonds (left), while viscoelastic materials usually contain weaker crosslinks such as ionic bonds (right) or physical entanglements that favour stress relaxation. **g** In viscoplastic materials, permanent deformations remain after the load is removed due to the breaking of bonds and the formation of new bonds. **h** During a creep test, viscoplastic materials do not return to their original shape and retain permanent deformations.

However, while most tissues appeared to be macroscopically solid materials, recent research has uncovered that their mechanical properties are significantly more complex^7,16^. Unlike purely elastic solid materials, tissues exhibit diverse mechanical behaviours with properties of both solids and liquids. In fact, all biological tissues and ECMs are viscoelastic^16–18^. Solid elastic materials deform instantly when subjected to a force and maintain a constant deformation as long as the force is applied, exhibiting time-independent behaviour (Fig. 1a, b). In contrast, liquid viscous materials undergo continuous and irreversible deformation when subjected to a force (Fig. 1a, b). Viscoelastic materials exhibit a response that lies between these two extremes. As viscoelastic materials, biological tissues initially respond elastically to a force and then undergo continuous viscous deformation. Due to this subsequent viscous response, viscoelastic materials will continuously deform with time while force is applied, a behaviour known as ‘creep’ (Fig. 1a–c). Similarly, when submitted to a constant strain, viscoelastic materials experience ‘stress relaxation’, meaning that stress relaxes with time (Fig. 1d). On the other hand, stress is constant over time in purely elastic materials (Fig. 1d). These behaviours emphasise the time-dependent nature of viscoelastic materials compared to the time-independent response of purely elastic materials.

The viscoelastic properties of tissues are largely determined by the mechanical properties of the ECM^16,18^. As a major regulator of tissues’ structural integrity, the ECM exhibits markedly higher mechanical strength compared to cells^19^. The ECM’s stiffness varies significantly, from a few hundred pascals in fat or brain tissue to gigapascals in bone^13,20^. Despite these differences in stiffness, all ECMs and tissues are viscoelastic^16^. The ECM viscoelasticity can be characterised using parameters such as the loss modulus and the stress relaxation half-time. The loss modulus quantifies energy dissipation during deformation and typically ranges from tens of pascals to gigapascals in the brain and bone, respectively^16^. Additionally, the stress relaxation half-time of tissues, which is the time that it takes for the stress to reduce to half of its original value under constant deformation, generally ranges between a few seconds in the brain to tens of minutes in skin or bone^16^. Examples of tissues whose viscoelastic properties have been measured include the brain^21–23^, breast^24^, bone^25,26^, skin^27,28^, bladder^29^, tendon^30^, kidney^31^, embryonic tissue^32^, liver^33–36^ and bone marrow^33,37^. In homoeostatic tissues, ECM viscosity typically accounts for about 10% of its stiffness^16^. The reason behind this linear relationship between stiffness and viscosity remains unknown. Deviations from this balance are associated with pathological conditions such as fibrosis, cancer or inflammatory diseases.

In the early 2010s, the development of novel materials with controlled viscoelastic properties accelerated the study of the influence of viscoelasticity in cell behaviour. Over the last decade, research has shown that ECM viscoelasticity can regulate various single-cell functions (for a more detailed review, see refs. ^16,17^) and collective cellular responses. More recently, the viscoelastic properties of the ECM have emerged as critical regulators of homoeostasis, morphogenesis and pathology. In this review, we present recent work highlighting the role of the viscoelastic properties of the ECM in development and disease.

## Viscoelastic properties of the ECM

The ECM is a complex scaffold made up of proteins and biopolymers, including fibrous collagen networks, laminins, hyaluronic acid, proteoglycans, and other large molecules^4,38^. These components offer critical physical and structural support to cells. The viscoelastic properties of the ECM in living tissues are influenced by these molecules and can be modulated by altering the proportion of each ECM component, their properties or the way they interact with each other. Since viscosity causes energy dissipation, any molecular event that results in energy loss will contribute to viscoelasticity. While energy is dissipated or lost in viscous materials, energy is stored in elastic materials.

One key factor regulating energy dissipation is the polymer molecular weight, which affects the movement of entangled polymers (Fig. 1e). Smaller molecules reduce the network’s interconnectivity and cohesion, thus promoting molecular mobility. These processes facilitate matrix flow under an applied force and, hence, viscoelasticity. The influence of molecular weight on cell response has been mainly studied using alginate^33,39–44^ and hyaluronic acid^45–47^, both viscous polysaccharides that create hydrogel structures. Another molecular mechanism that contributes to ECM viscoelasticity is the strength of the bonds that crosslink the ECM (Fig. 1f). Stress relaxation is favoured in weakly crosslinked ECMs, such as those linked ionically or physically, but is prevented in strongly crosslinked ECMs, such as those linked covalently. In networks formed by collagen or fibrin fibres, these fibres are primarily crosslinked by weak bonds. These weak bonds facilitate fibre displacement and energy release under force, enabling matrix relaxation or creep. Weak bonds can respond to force by dissociating and then rebinding, which may lead to plastic or permanent deformations (Fig. 1g). Materials experiencing permanent deformations are viscoplastic and represent a subset of viscoelastic materials.

**Fig. 2:**
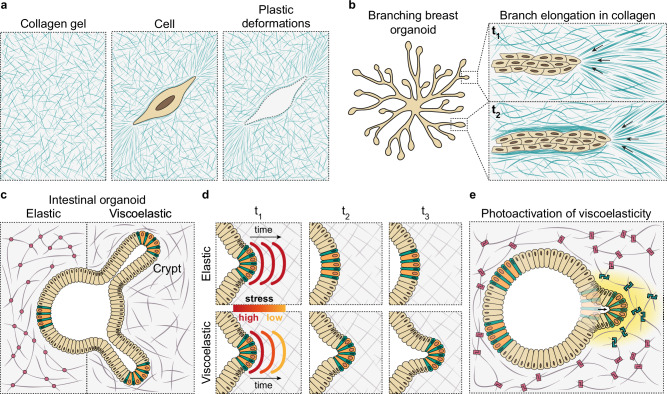
The viscoelastic properties of the ECM regulate tissue response during development. **a** Collagen fibres can be realigned by cells in the direction of the deformation, resulting in permanent, plastic deformations. **b** Breast organoids cultured in collagen gels undergo branching morphogenesis. Branch extension is mediated by plastic deformation of the matrix due to the cyclic pulling of cells on the matrix (t_1_). This process leads to the alignment of collagen fibres in front of the branch, resulting in a strong enrichment of aligned collagen fibres along the branch as it extends^111^ (t_2_). Black arrows represent the direction of the strain. **c** Intestinal organoids are unable to break symmetry in elastic matrices and grow as cysts. In contrast, intestinal organoids cultured in viscoelastic matrices form crypt-like structures containing stem cells (green) and Paneth cells (orange)^43^. **d** In purely elastic matrices, when a group of cells applies force to deform the matrix, the matrix resists with an equal force (top). In viscoelastic matrices, however, stresses gradually relax over time, facilitating cell groups to maintain the deformation and proceed with morphogenesis (bottom). **e** In an elastic matrix with photoinducible increase in viscoelasticity, a local increase in matrix viscoelasticity (yellow) increases intestinal organoid curvature and membrane tension, leading to YAP nuclear translocation and symmetry breaking^53^.

Plastic deformation occurs when a material is subjected to stresses beyond a threshold known as yield stress, which results in a permanent change in shape once the stress is removed^7^ (Fig. 1h). For instance, when collagen is significantly strained by cells, cells are able to generate plastic deformations and align collagen fibres in the direction of the deformation^48^ (Fig. 2a). This strain promotes the local stiffening of collagen fibres^48^, a phenomenon known as strain stiffening^49^. Recent studies have also shown that the alignment of collagen fibres leads to a local increase in the rate of stress relaxation, thereby enhancing viscoelastic properties in those areas^50^. Even though the native ECM is mainly crosslinked with weak bonds, these bonds coexist with strong covalent bonds, as crosslinked with lysyl oxidase. Covalent bonds prevent the matrix from flowing and dissipating energy, thereby promoting a predominantly elastic behaviour of the material^33^ (Fig. 1f). These elastic matrices are unable to relax and plastically deform. In vitro, synthetic matrices are typically crosslinked with covalent bonds and, hence, behave as purely elastic materials. However, recent advancements have led to the development of tuneable synthetic hydrogels with dynamic bond rearrangements, resulting in viscoelastic matrices^51–55^. An alternative approach to creating viscoelastic hydrogels is to incorporate linear polyacrylamide, a dissipative component, into the structure of covalently crosslinked polyacrylamide hydrogels^56^.

Tissues are primarily composed of water, and variations in water content significantly impact the viscoelasticity of the ECM. Poroelasticity describes the behaviour of materials with fluid-filled pores under mechanical stress. When pressure is applied, water flows in or out of the ECM’s porous network, leading to a time-dependent response due to changes in volume. These changes cause viscous dissipation, which impacts the ECM’s viscoelastic properties. Polysaccharides such glycosaminoglycans retain water, regulating fluid flow and ECM’s viscoelastic properties^23,57^.

## Cell response to viscoelasticity

The viscoelastic properties of the natural ECM have been recognised for some time, but their influence was not well understood until recently^16,17,58,59^. The development of novel materials that enable the independent control of the matrix viscoelastic properties has allowed researchers to study the function of ECM viscoelasticity^60^. The most commonly utilised engineered matrices are polyacrylamide^56,61^, alginate^18,33,55^ and polyethylene glycol (PEG)^51,53^ hydrogels. Taking advantage of these materials, studies have shown that the matrix stress relaxation, creep or plasticity regulate cell behaviour. Substrate viscoelasticity regulates two-dimensional (2D) cell spreading^41,56,62–65^, migration^66^, focal adhesion growth^41,56,64^, stress fibres formation^41^ and yes-associated protein 1 (YAP) nuclear translocation^41^. Similarly, in purely viscous substrates composed of lipids, increasing viscosity favours cell spreading, focal adhesion reinforcement, and YAP nuclear localisation^67^. These responses triggering mechanotransduction are similar to how cells react to an increase in stiffness in 2D environments. In fact, a similar mechanosensitive mechanism, known as the molecular clutch model, has been proposed to regulate both stiffness and viscoelasticity sensing^16,68^. In this model, myosin pulls on the actin filaments towards the nucleus, generating an actin rearward flow. The actin rearward flow is resisted by integrins that bind to the ECM. Integrins attach to the ECM with a given binding rate and an unbinding rate dependent on the force that is applied to them^69^. This model also suggests potential differences in mechanosensing due to the time-dependent behaviour of viscoelastic materials versus the time-independent response of elastic materials^41,63,66^. In an elastic material, for a given deformation and stiffness, clutches are subjected to the same forces regardless of time^70,71^. However, in viscoelastic matrices, these forces relax over time. These substantial changes affect the apparent stiffness that cells perceive, which diminishes with time. Theoretical modelling and experiments show that the relationship between the characteristic stress relaxation time of a material and the integrin unbinding rate regulates cell spreading^63^. Maximum spreading occurs when the relaxation time falls between the timescale of clutch binding and the characteristic clutch lifetime^63^. This research suggests that the differences between viscoelastic and elastic materials are most pronounced at low stiffness. More research is required to validate and understand the potential differences between elasticity and viscoelasticity sensing.

The influence of the ECM viscoelasticity in cell response is more complex in three dimensions than in two dimensions. Besides stiffness and viscoelasticity, the microarchitecture of the ECM plays a crucial role in three-dimensional (3D) cell migration. In 3D environments, cells must navigate through intricate surroundings and small pores, which can hinder their ability to migrate. Cell migration in 3D confined spaces is constrained by the size and stiffness of the nucleus, which makes it challenging to move through small pores. Cells cannot typically squeeze through pores smaller than 7 μm^2^ without the degradation of the surrounding matrix^72^. However, recent research with nanoporous materials has shown that cells are able to physically deform pores in viscoelastic materials to migrate without the need for proteolysis^73^. While cells are unable to migrate in nanoporous slow-relaxing gels, cells in fast-relaxing gels migrate forming long, shallow protrusions^73^. The nucleus is pushed towards the protrusions, functioning as a piston, which facilitates cell migration^74^. This process is regulated by the actin cytoskeleton, microtubules, intermediate filaments and the transient receptor cation vanilloid (TRPV4) and Na+/H+ exchanger (NHE1) ion channels^74^. The ability of cells to generate space in viscoelastic matrices^43,75,76^ not only favours cell migration but also single-cell spreading^33,54^, cell division^77^, cell cycle progression^78^, differentiation^33,62,79^, matrix deposition^33,80^ and bone formation and regeneration^33,81^. Cell-driven ECM remodelling can modify pore size and alter the ECM’s viscoelastic properties, influencing cell migration. For instance, previous research with PEG hydrogels with degradable crosslinks showed that cellular degradation during migration transforms the initially elastic hydrogel into a viscoelastic fluid^82^.

Although collective cell responses to ECM viscoelasticity have not been studied as extensively as single-cell responses, research has shown a significant impact in 3D environments, both in spheroids and organoids. In elastic matrices, spheroids tend to grow slowly and maintain their spherical symmetry^43,75,76,83^, however, viscoelasticity promotes symmetry breaking, branching and migration^43,76^. This response is regulated by cell migration and proliferation^43^. Similarly, in organoids, matrix viscoelasticity supports self-organisation, promoting morphogenesis and patterning^42,43,53,84^.

## The influence of matrix viscoelasticity in development

Successful tissue development relies not only on proper cellular arrangement but also on the acquisition of appropriate structural forms, with the ECM serving as a primary contributor to this process^85^. The precise patterning and organisation of tissues are crucial for the development and functionality of all organisms. Alongside their patterning, tissues need to acquire the correct shape through morphological transformations such as folding or buckling. The viscoelastic properties of the ECM have recently emerged as a critical parameter that regulates many developmental processes ranging from embryonic development to organ morphogenesis.

### Embryonic morphogenesis

During embryonic development, ECM remodelling is necessary and will inherently change the mechanical properties of the ECM. In the mouse epiblast, Nodal, a signalling molecule that directs cell fate decisions, regulates matrix metalloproteinase (MMP) expression and, in turn, MMP-dependent basement membrane perforations allow for embryo growth due to local basement membrane stress relaxation^86^. Perforations increase matrix pore size, which accelerates stress relaxation^87^. Following the specification of the anterior-posterior axis, where the head-to-tail orientation is established, the asymmetric Nodal gradient restricts basement membrane perforations to the posterior side, facilitating the extension of the primitive streak and gastrulation^86^. In vitro, human induced pluripotent stem cells (hiPSCs) can form lumens^44^ with a morphology similar to the epiblast, but only when cultured in viscoelastic hydrogels^88^. The lumen formation occurs due to apical actin polymerisation and osmotic pressure^88^. In Drosophila, the viscous properties of the basement membrane regulate the central nervous system morphogenesis. Upon collagen IV assembly, the basement membrane flows viscously around the ventral nerve cord toward the embryo’s anterior side. This flow precedes cell migration and enhances surface tension along the tissue, leading to ventral nerve cord condensation^89^. During wing morphogenesis, the viscoelastic properties of the tissue control its shape and size. The wing tissue shapes itself through patterned contractions and shear, regulated by the patterned extracellular connections to the cuticular scaffold. These connections force the tissue into shape by controlling surface tension and controlled tissue flow through the regulation of cell size, division and extrusion^90^.

Beyond ECM properties, much research is currently dedicated to studying the rheology of cells and groups of cells during embryonic development^91^. Although viscoelastic measurements of embryonic cells or tissues have existed since the late 1990s^32^, it is only recently that the influence of cellular and collective viscosity has started to be thoroughly investigated, showing in Xenopus embryos that the gastrulating blastula behaves as a viscoelastic material^92^. Recent novel measurement techniques have shown that tissue viscoelastic properties vary during development, suggesting a potential mechanism to regulate morphogenesis^93–97^. Recent research has shown that morphogens can modify tissue material properties^95,98,99^. For example, while Nodal fluidises tissue during mesoderm internalisation in zebrafish gastrulation^98^, Wnt prevents fluidisation during blastoderm expansion^95^.

Tissue-scale viscoelastic properties are usually defined by tissue architecture and specific cellular features. Solid-like behaving tissues are often observed in densely packed, symmetrical cell shapes, high cell-cell adhesion and low cell motion tissues. In contrast, liquid-like behaving tissues are often associated with low density, asymmetric cell shapes, low cell-cell adhesion, frequent cell rearrangements and random or significant cell motion^100–104^. Most of these studies qualitatively infer tissue fluidity by quantifying cell topology, density or motility, as accurately determining the precise viscous and elastic properties of cells is a challenge. However, there are several quantitative techniques to infer the viscoelastic properties of tissues. Some of these techniques are the same as used to characterise inert materials and ECMs, such as atomic force microscopy or rheometry, and others are specifically utilised to characterise live tissues, such as micropipette aspiration or ferromagnetic droplets. These methods can provide key parameters such as viscosity, loss modulus or stress relaxation. For instance, the Campàs lab has recently developed ferromagnetic droplets to be able to provide quantitative measurements of viscoelasticity during the elongation of the presomitic mesoderm in zebrafish and demonstrated a continuous reduction in tissue viscoelasticity during elongation^93,94,105^. The authors were able to perform a similar quantitative analysis of the values of viscoelasticity as in material science, adding significantly more relevance to these results.

### Branching morphogenesis

Significant research has focused on understanding the influence of the ECM on branching morphogenesis^106^, the process by which epithelial branched tubular structures form in various organs, including lung, breast, pancreas, kidney and salivary glands^106^. Research conducted on avian embryos has shown that during lung development, an increase in the mesenchyme ECM fluidity promotes lung branching morphogenesis^107^. This increase in ECM fluidity, caused by the expression of MMP2, is necessary for branch extension but not for branch initiation^107^. Theoretical modelling, combined with experiments with lung explants, have shown that branch initiation occurs due to the viscoelastic properties of the matrix, in the absence of a biochemical template^108^. The proliferation of lung epithelium can create a mechanical instability regulated by the viscoelastic properties of the ECM, leading to branch formation^108^. Branching morphogenesis of the mammary gland occurs through different mechanisms at three distinct stages: embryonic, pubertal, and adult, leading to the formation of a full epithelial tree. The mammary fat pad is relatively large and not easily accessible optically, which makes imaging more difficult. Due to these limitations, breast branching morphogenesis dynamics have been studied in vitro with breast organoids by embedding them in a scaffold with a defined concentration of two natural viscoelastic materials: Matrigel and collagen I^109^. Matrigel (or similar commercially available products such as Geltrex), is an animal-derived matrix composed of more than two thousand proteins^110^. This scaffold allows breast organoids to recapitulate some of the critical developmental processes that occur during puberty and reproductive cycles^109^. Buchmann et al. showed that the plasticity of collagen I favours branch elongation in human mammary organoids^111^ (Fig. 2b). These permanent deformations in the collagen network occur due to the pulling force of branch tip cells on collagen fibres and the back-and-forth movement of epithelial cells within the branch. This process leads to highly anisotropic plastic deformations and the formation of highly aligned fibre bundles along the branch elongation axis^111^ (Fig. 2b). These collagen fibres form a mechanically stable collagen cage that encloses the elongating branch, thereby confining and coordinating cell movement and thus guiding branch growth^111^. Moreover, the increase in stiffness due to local ECM accumulation directs the bifurcation angle of mammary terminal end buds^112^.

During kidney development, branching morphogenesis and nephrogenesis, which take place at the branch nodes, occur simultaneously. While both processes are critical for kidney morphogenesis, the influence of the ECM’s mechanical properties has been primarily studied in the context of nephron development. Nephron formation has been mainly studied in vitro with human pluripotent stem cells (hPSCs)^42,113,114^. For example, previous research with hPSCs-derived organoids cultured on 2D purely elastic substrates has shown that stiffness regulates kidney organoid development. Soft substrates favour glomerulus and tubule-like structures and express higher levels of late-stage nephron marker^113^. While 2D substrates favour development, hiPSCs-derived kidney organoids encapsulated in 3D stiff elastic hydrogels express epithelial to mesenchymal transition (EMT) markers, observed in early renal fibrosis^114^. Nevertheless, encapsulation in soft fast-relaxing gels prevents EMT as well as promotes apical proximal tubule polarisation and primary cilia formation^114^. Furthermore, fast stress relaxation favours a broader distribution of nephron segments in the organoids. As to nephrogenesis, recent research with hPSCs-derived kidney organoids on viscoelastic alginate hydrogels has shown that, while stiffness does not play a role, matrix viscoelasticity regulates nephron segments spatial distribution^42^. Apart from viscoelasticity, 3D confinement promotes the formation of more convoluted proximal and distal tubule nephron segments, closer to in vivo kidney morphogenesis^42^.

### Intestinal development

The impact of matrix viscoelasticity on organ development has been studied more extensively in intestinal organoids than in other organoids. The intestine is one of the best paradigms for tissue patterning that is accompanied by morphological changes, specifically folding. For over a decade, intestinal organoids have been used to mimic crypt development in vitro as they can self-organise spatially and temporally, forming crypt-like and villus-like structures^115^. Organoids are typically embedded in Matrigel or similar, which have poorly defined and complex biochemical and viscoelastic properties^110^. Significant efforts have been made to understand the role of Matrigel mechanics, and of the ECM mechanics in general, in organoid growth. Consistent results have shown that organoids are unable to break morphological symmetry in 3D purely elastic PEG^14,116–118^ or 2D polyacrylamide hydrogels^119^ (Fig. 2c, d). For organoids to self-organise in PEG elastic materials, rapid matrix degradation is necessary^14,120^, leading to progressive changes in mechanical properties, including an increase in porosity and a decrease in stiffness. Unlike purely elastic materials, matrix viscoelasticity enables intestinal organoid morphogenesis and crypt formation^43,52,53^ (Fig. 2c, d). Indeed, the relaxation of stress in viscoelastic matrices allows groups of cells to deform the matrix, as observed in intestinal organoid folding (Fig. 2d). For instance, a photoinduced local increase in viscoelasticity due to bond rearrangements in a purely elastic PEG hydrogel supports symmetry breaking by enhancing tissue curvature, leading to an increase in membrane tension and YAP nuclear localisation^53^ (Fig. 2e). YAP nuclear localisation has been shown to be involved in symmetry breaking of intestinal organoids^121^, and is indeed observed in organoids grown both in Matrigel and PEG hydrogels with fast stress relaxing properties^52,53^. Using alginate-based viscoelastic hydrogels, organoids can self-organise into crypt and villus-like regions, where cells differentiate into various intestinal lineages, including enterocytes, Paneth cells, enteroendocrine cells, goblet cells, and stem cells^43^. Theoretical modelling and experiments have shown that fast stress relaxation facilitates collective cell movement and proliferation, leading to the development of high curvature regions and crypt formation^43^. Apart from the ECM viscoelasticity, recent research has shown that mesenchymal cell fluidisation initiates villi formation in vivo^122^. The aggregation of fibroblasts with liquid-like material properties leads to the bending of the interface, which initiates villi formation^122^.

## The influence of the ECM viscoelasticity in disease

A significant number of diseases involve pathological ECM remodelling, which influences disease progression and clinical outcomes^123^. Most research in this area has concentrated on how stiffness affects disease, as ECM remodelling often leads to tissue stiffening. However, less attention has been given to ECM viscoelastic properties. Studies indicate that disruptions in the ECM viscoelastic properties affect immune responses^124^, and contribute to various pathological conditions, including cancer^73^, chronic inflammatory diseases^125^, and neurological disorders^21^. Understanding both stiffness and viscoelasticity is essential for a comprehensive view of how ECM changes influence disease progression.

### Cancer

**Fig. 3:**
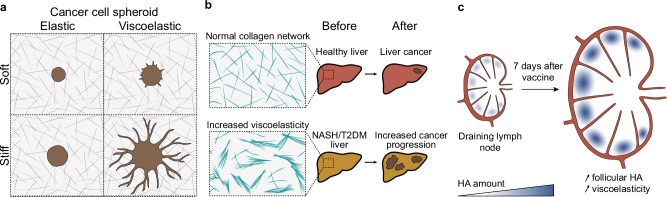
Distinct cellular responses to viscoelasticity in disease. **a** Cancer cell spheroids cultured in purely elastic matrices grow but are unable to invade regardless of matrix stiffness. In viscoelastic matrices, cancer cells are able to break symmetry, undergo epithelial to mesenchymal transition and migrate into the matrix. Viscoelasticity and stiffness seem to have a synergistic effect, and these behaviours are enhanced in stiff, viscoelastic matrices^43^. **b** The liver of non-alcoholic steatohepatitis (NASH) and type 2 diabetes mellitus (T2DM) patients has a similar stiffness but an increased viscoelasticity compared to healthy patients. This increase in viscoelasticity is due to the accumulation of advanced glycation end-products affecting the structure of the ECM, decreasing collagen fibre length and network homogeneity. This increase in viscoelasticity favours hepatocellular carcinoma progression^36^. **c** Following vaccination, lymph node expansion is accompanied by an increase in viscoelasticity and a strong hyaluronic acid (HA) enrichment in the follicles. The immune response efficacy scales with lymph node expansion^149^.

Cancer encompasses a variety of heterogeneous diseases whose progression and regression are strongly influenced by the tumour microenvironment^126^. This environment is often marked by a pathologically remodelled ECM, and the frequent stiffening of the ECM from this remodelling is a key feature of several tumours^7,123^. Stiffness is often used as a cancer biomarker and palpation or imaging techniques are used to detect ECM remodelling. Nonetheless, this ECM remodelling likely modifies ECM viscoelasticity, and how these variations in viscoelasticity affect cancer progression is unknown. The development of new methods has advanced the ability to measure changes in the viscoelastic properties of cancer^127–130^. This progress is largely due to the growing focus on measuring the viscoelastic properties of tumours as potential disease biomarkers. Studies suggest that tumour matrix viscoelasticity could be used for diagnosis, as different malignant tumours have characteristic viscoelastic properties, which are different to healthy tissues. Despite these characteristic signatures, research has highlighted that there is no universal rule; some tumours are more viscoelastic, while others are more elastic than healthy tissue. Breast tumours, as well as liver tumours and metastases, are significantly stiffer but also more viscoelastic than normal tissue^24,130–132^. These results suggest that taking into account both viscoelasticity and stiffness may provide the necessary information to detect and mechanically characterise each cancer. Multidisciplinary approaches combining theory and experiments have shown that stiffness and viscoelasticity may play different but complementary roles^36,43,133^. Recent research has shown that matrix viscoelasticity triggers breast spheroids growth, symmetry breaking, invasion and EMT both in vitro and in vivo^43^ (Fig. 3a). Interestingly, when the matrix is viscoelastic, stiffness has a synergistic effect and promotes cancer growth and invasion (Fig. 3a). This behaviour is regulated by cell migration and proliferation in a mechanism regulated by FAK–Arp2/3 complex–Rac1^43^. Moreover, Wisdom et al. showed that mechanical plasticity also promotes cancer cell migration in nanoporous matrices in a protease-independent migration mode. Cells can deform and widen the matrix pores by exerting forces, extend protrusions and migrate in a 3D matrix^73^. Similarly to breast cancer, increased liver ECM viscoelasticity promotes hepatocellular carcinoma progression^36^. The fact that type 2 diabetes mellitus and non-alcoholic steatohepatitis livers are more viscoelastic favours hepatocellular carcinoma progression^36^ (Fig. 3b). This increase in viscoelasticity is caused by ECM microstructural changes due to the accumulation of advanced glycation end-products (Fig. 3b). These changes decrease collagen fibre length and increase network heterogeneity, thereby raising ECM viscoelasticity^36^. This increase in viscoelasticity favours invasion and cell proliferation through an integrin β1–tensin-1–YAP dependent mechanism^36^. Additionally, viscoelasticity can be used to discriminate between diseases. For example, pancreatic ductal adenocarcinoma is less viscoelastic than pancreatitis^134^, and glioblastoma is less viscoelastic than meningioma^23,135^. In fact, it has been suggested that the higher viscosity of glioblastoma may generate Saffman-Taylor instabilities leading to viscous fingering of glioblastoma cells into the surrounding tissue, which could enhance its invasive potential^23^. More research is needed to clarify the role of ECM viscoelasticity in cancer response.

Apart from the ECM viscoelasticity, recent evidence has suggested that the extracellular fluid viscosity can favour breast cancer cell migration. Increased fluid viscosity enhances NHE1 polarisation via the Arp2/3 complex^136^. NHE1 induces cell swelling and membrane tension, which activates TRPV4 regulating calcium influx and contractility^136^. This mechanism boosts cell migration in the presence of high extracellular fluid viscosity. Moreover, cancer cells transiently exposed to high extracellular viscosity retain an enhanced motility, extravasation and lung colonisation^136^.

In addition to the extracellular microenvironment, the viscoelastic properties of cancer cells can also regulate cancer progression^137,138^. Research has demonstrated that cancer cells have greater fluidity compared to healthy cells^137,139,140^. These results imply that single cell viscosity could serve as a biomarker for cancer detection. Apart from single cells viscoelasticity, tumours’ multicellular fluidity has been shown to increase in several cancers^135,141,142^. Enhanced tissue fluidity allows cells to move and rearrange more easily within tissues, promoting cancer cell migration and invasion. This transition from solid-like behaviour to fluid-like behaviour occurs during EMT due to a decrease in the stability of cell-cell adherens junctions^143^, such as E-cadherin and p120-catenin, and is also regulated by the degree of ECM confinement^144^. Besides EMT, tissue fluidisation and invasion can also be driven by the overexpression of the small GTPase Rab5A^145^. This overexpression increases the endocytosis of the EGF receptor and activates ERK1/2, leading to branched actin polymerisation. As a result, cancer cells engage in collective migration through cryptic lamellipodia^145^. Recent research has suggested that tissue fluidity may be heterogeneous inside tumours. Tumours may contain regions of stiff solid-like immobile cells surrounded by soft fluid-like motile cells^146^. The heterogeneous distribution of stiff cell clusters creates a network of tension across the entire tumour. This tension network percolates through the softer, unjammed regions, allowing the tumour to exhibit a mechanically stable, solid, and stiff bulk behaviour. This occurs without needing a large proportion of stiff cells or a continuous network of interconnected stiff cells^146^. This network may explain why cancer cell migration is not only restricted to the tumour edges, and cancer cells can also actively and coordinately migrate in the tumour core^147^.

### Immune response

The ECM viscoelasticity can also influence the immune system’s response. Adu-Berchie et al. showed that collagen networks’ viscoelasticity affects T cell phenotype both in vitro and in vivo^124^. While collagen matrices with low viscoelasticity increased T cell cytotoxicity, highly viscoelastic matrices increased T cell memory markers, implying that tissue localisation could impact T cell phenotype^124^. These findings have direct implications for T cell function and immunotherapies in vivo, as many tumours exhibit high viscoelasticity. Besides T cells, monocytes also respond to the viscoelastic properties of the ECM through PI3K-gamma and cortical F-actin^148^. In viscoelastic matrices, monocytes retain an immature phenotype, whereas in elastic matrices, they differentiate into dendritic cells and fibrocytes. This behaviour mimics the abnormal differentiation seen in myelofibrosis^148^. Significant changes in lymph node viscoelasticity have also been observed after vaccination, correlating with an increase in hyaluronic acid expression^149^. Following vaccination, hyaluronic acid is enriched in the follicles, and lymph node expansion increases its viscoelasticity, leading to a rise in inflammatory monocytes^149^ (Fig. 3c). This expansion is correlated with the magnitude and efficacy of the immune response^149^.

### Chronic inflammatory diseases

Since ECM remodelling is crucial in chronic inflammatory diseases, research has explored the impact of ECM viscoelasticity on chronic inflammation. Chronic inflammation can affect multiple organs, including the lungs, liver, gastrointestinal tract, joints, and nervous system. For example, healthy chondrocytes enhance matrix production in viscoelastic matrices, whereas less viscoelastic matrices trigger an inflammatory state, due to the cell volume restriction caused by confinement^80^. However, osteoarthritis chondrocytes cannot respond to matrix viscoelasticity due to a defective TRPV4–GSK3β mechanotransduction axis, leading to a chronic inflammatory phenotype^150^. Inflammatory bowel disease results in intestinal mucus that is stiffer and more viscoelastic than normal. Increased mucin secretion and abnormal glycosylation are responsible for this change, which increases inflammation and makes the mucus more penetrable by bacteria^151–154^. In many chronic inflammatory diseases, fibrosis is a prevalent outcome. Fibrosis is characterised by an excessive buildup of ECM by myofibroblasts in response to tissue injury and inflammation, particularly of collagen I and elastin, as well as higher levels of collagen-crosslinking lysyl oxidase enzymes. This results in an increase in ECM stiffness and viscoelasticity, as observed for instance in hepatic fibrosis, pulmonary fibrosis and intestinal fibrosis^125,155–159^.

### Neurological disorders

Diseases of the central nervous system are an increasing concern in aging populations and are still challenging to detect at early stages. Interestingly, there is already strong evidence of altered brain mechanics in neurological diseases. For example, studies have shown that the brain becomes softer and more viscoelastic with age-related brain atrophy^22,160^. In patients with multiple sclerosis, brain viscoelasticity is reduced compared to healthy controls, although stiffness remains unchanged^21,161^. This reduction might be linked to the accumulation of high molecular weight hyaluronic acid in demyelinated regions^162^. Viscoelasticity is also reduced in Alzheimer’s disease^163,164^. This decrease in viscoelasticity may be linked to an increase in hyaluronic acid content and the hyaluronic acid crosslinker TSG-6 during the progression of the disease^165,166^. Additionally, these studies indicate gender differences in brain mechanical properties, with female brains being stiffer and more viscoelastic than male brains^22,160,161^. Given that women are statistically more likely to develop conditions such as multiple sclerosis and Alzheimer’s disease, further investigations into these differences could provide valuable insights.

## Conclusions and perspectives

All extracellular matrices and tissues exhibit viscoelastic properties, resulting in complex, time-dependent responses to forces and deformations. Past and ongoing development of novel materials with controlled properties, that more closely mimic the natural ECM viscoelasticity, has significantly enhanced our understanding of how viscoelasticity regulates cell and tissue behaviour. Over the past decade, studies have demonstrated the impact of ECM viscoelasticity on single-cell responses^16^, challenging the prevailing stiffness-centric view of mechanobiology. More recently, research has highlighted its potential effects on collective tissue morphology, dynamics and organisation^36,43,44,53,107^. Despite these advances, our understanding of viscoelasticity remains superficial. The time-dependent properties of ECMs and tissues are intricate, and unravelling the mechanisms by which different levels of viscoelasticity influence single and collective cell responses is challenging. While the molecular mechanisms by which cells sense the viscoelastic properties of 2D substrates are relatively well understood^16^, how cells sense and respond to 3D matrices remains less clear and significantly more complex. In 3D ECMs, cell sensing and response are influenced by both confinement and time-dependent viscoelasticity. The dynamic nature of viscoelasticity leads to continuous changes in confinement and stresses, which in turn actively impacts cell behaviour. The regulation of this feedback mechanism over time is still not understood. New tools and methods are required to improve our mechanistic understanding of mechanotransduction in 3D viscoelastic matrices.

Another critical and unresolved question is understanding how ECM viscoelasticity interacts with stiffness to control tissue dynamics. Recent research suggests that viscoelasticity and stiffness play synergistic yet distinct roles^33,36,43^, with viscoelasticity potentially having a more dominant influence^43^. However, to better understand the interplay between viscoelasticity and stiffness, it is crucial to measure the viscoelastic properties of tissues during development, as well as in adult and pathological states. Different studies have shown that while increased stiffness is associated with various diseases, there is no clear trend regarding how viscoelasticity affects disease. Some tumours exhibit increased viscoelastic properties^24,130–132^, while others decrease them^23,134,135,141^. Consequently, the extent to which viscoelasticity influences tissue response remains unclear. These findings suggest that we may be overlooking critical factors related to tumour evolution and how the ECM impacts tissue response. Additionally, these studies also indicate that several diseases can be distinguished by considering both stiffness and viscoelasticity^23,134,141^, highlighting the importance of evaluating both mechanical properties.

At the multicellular level, tissues are continuously changing within 3D viscoelastic matrices, but how the viscoelastic properties adapt to the tissues and evolve dynamically is not yet understood. While some examples suggest that ECM viscoelasticity regulates various processes during development and disease, it remains unclear whether viscoelasticity affects tightly regulated processes, such as morphogenesis, differently than it does more uncontrolled processes like cancer. To gain a better understanding of the role of viscoelasticity in development and disease, it is crucial to explore how cells interacting with the ECM convey its viscoelastic properties to the rest of the tissue, as well as the physical and biochemical consequences of ECM viscoelasticity. This would provide valuable insights into its influence on these processes. Addressing these complex questions requires a multi-scale approach that integrates molecular biology, cell mechanics, theoretical modelling, in vivo experiments, and biomaterials engineering. Delving into the effects of viscoelastic properties on tissue dynamics during development and diseases is an exciting and essential area of research for both today and the future.

## Data Availability

No datasets were generated or analysed during the current study.
